# Polymeric Carriers for Biomedical and Nanomedicine Application

**DOI:** 10.3390/polym13081261

**Published:** 2021-04-13

**Authors:** Sofia A. Costa Lima, Salette Reis

**Affiliations:** LAQV, REQUIMTE, Departamento de Ciências Químicas, Faculdade de Farmácia, Universidade do Porto, Rua de Jorge Viterbo Ferreira, 228, 4050-313 Porto, Portugal

Polymeric carriers play a key role in modern biomedical and nanomedicine applications. Polymers can be obtained from natural or synthetic sources and have been exploited given their chemistry to achieve interaction with living tissues and cells. Different types of carriers can be produced for drug delivery, namely, micelles, nanoparticles, dendrimers, sponges, hydrogels, and microneedles. With different coatings, appropriate adhesion and targeting features can be designed. Polymeric carriers allow the incorporation or conjugation of both hydrophilic and hydrophobic molecules and have tunable chemical and physical features that allow effective drug protection from degradation or denaturation. Other features are noteworthy in polymeric carriers, like their generally good biocompatibility and the ability to exhibit a slow and controlled dug release, allowing for the use in biomedical applications.

This Special Issue provides an encompassing view on the state of the art of polymeric carriers, showing how current research is dealing with new stimuli-responsive systems for cancer therapies and biomedical challenges, namely, overcoming the skin barrier. The published papers cover topics ranging from novel production methods and insights on hybrid polymers to applications as diverse as nanoparticles, hydrogels, and microneedles to antifungal skin therapy, peptide and siRNA delivery, enhanced skin absorption of bioactive molecules, and anticancer therapy. This Special Issue contains one review paper on modulation of macrophage polarization mediated by carbohydrate-functionalized polymeric nanoparticles [1]. A couple of polymeric carriers targeting macrophages have been reviewed in terms of production methods and conjugation approaches. The role of mannose receptor in the polarization of macrophages is highlighted as strategies for infectious diseases and cancer therapies as well as prevention actions.

Taking advantage of polysaccharides’ physicochemical features, Pontillo et al. designed new biocompatible and cost-effective carriers for tyrosol, a bioactive natural product present in olive oil and white wine [2]. A chitosan based nanosystem was obtained using the ionic gelation method, while for β-cyclodextrin (βCD), the kneading method was employed. Additionally, coating of the tyrosol–βCD inclusion complex with chitosan led to a sustained release of tyrosol and slowed down the initial burst effect observed from the inclusion complex. The nanosystems were extensively characterized after optimized production based on a two- or three-factor, three-level Box–Behnken experimental design. Moreover, the interaction of tyrosol and the corresponding nanosystems with ctDNA was evaluated. Data suggest that tyrosol is a ctDNA groove binder, which was confirmed by molecular modeling studies. The same mode of binding was found only for the tyrosol/βCD and tyrosol/βCD/chitosan nanosystems. Nanocomposites of chitosan and alginate were exploited by Sabbagh et al. to deliver metronidazole [3]. Optimization of the formulation was obtained using a full factorial design to study the effect of chitosan and alginate polymer concentrations and calcium chloride concentration on drug loading efficiency, particle size, and zeta potential. These dependent variables were affected by the chitosan, alginate, and calcium chloride concentrations, while zeta potential depended only on the alginate and calcium chloride concentrations. The applied mathematical models revealed that the developed response surface methodology models were statistically significant and adequate for all conditions. High correlation values were determined between the experimental data and predicted ones. The optimized nanocomposites were physiochemically characterized by X-ray diffraction, Fourier-transform infrared spectroscopy, thermal gravimetric analysis, scanning electron microscopy, and in vitro drug release studies. Overall, the optimized nanocomposites could be effective in sustaining the metronidazole release for a prolonged period. Hybrid nanosystems have been studied by Duskey et al. to increase the applicability of poly(lactic-co-glycolic acid) (PLGA) in drug delivery [4]. A series of unique PLGA–chitosan hybrid polymers with tailored and tunable physicochemical characteristics were obtained with two different synthetic methods: solid-phase synthesis on a film or in solution chemical reaction with polycaprolactone as intermediate. The hybrid polymers were physiochemically characterized using nuclear magnetic resonance, Fourier-transform infrared spectroscopy, and dynamic scanning calorimetry. A sodium dodecyl sulfate (SDS) salting-out reaction led to a chitosan SDS intermediate that is soluble in organic solvents, and consequently, a new series of PLGA–chitosan copolymers with different molar ratios were produced. The unique series of PLGA–chitosan hybrids with various molar rapports and solubilities represent the expansion of the PLGA delivery system for the protection and delivery of a wide range of previously noncompatible drugs either as nanoparticles formed through chitosan self-assembly techniques (for those still soluble in acidic solutions) or for the encapsulation in stable and nontoxic films for long-term controlled release (for those insoluble in biological solutions). 

Shin et al. developed PLGA nanoparticles as siRNA carriers to overcome ROS/oxidative stress-induced chondrocyte damage in osteoarthritis [5]. A double emulsion technique allowed the successful incorporation of siRNA p47phox within PLGA nanoparticles. The nanosystem was physicochemically characterized and evaluated in chondrocytes and in an osteoarthritis in vivo model. The formulated PLGA nanoparticles provided a sustained release of siRNA, which could reduce dosing frequency to a weekly regimen. Inhibition of p47phox by nanoparticles delivered siRNA-attenuated pain behavior, cartilage damage, and ROS production in knee joints with induced osteoarthritis. The developed polymeric nanosystem may represent a promising novel therapeutic avenue for the treatment of osteoarthritis. PLGA nanoparticles were explored for peptide delivery by Lima et al. [6]. A peptide from the myeloid proteolipid protein (PLP) was encapsulated in PLGA nanoparticles and further incorporated within polymeric microneedle patches for an effective skin delivery. Trehalose was included to preserve the nanoparticles during the freeze-drying process. Polydimethylsiloxane molds were used to obtain poly(vinyl alcohol)–poly(vinyl pyrrolidone) microneedles to carry the freeze-dried PLP-loaded PLGA nanoparticles. Microneedle patches with 550 µm height and 180 µm diameter allowed the peptide release in physiological media. The achieved outcomes motivate the exploitation of this strategy as a new antigen-specific therapy, providing minimally invasive administration of PLP-loaded nanoparticles into the skin. Struggling with skin drug delivery, Zhang et al. studied the effect of poly(ethylene glycol) (PEG) 400 and PEG-6-caprylic/capric glycerides on the dermal absorption of niacinamide [7]. Binary and ternary systems composed of PEGs or PEG derivatives combined with other solvents were studied for skin delivery of niacinamide. Porcine skin permeation assays over 24 hours revealed improved performance of all designed vehicles in relation to PEG 400. High skin retention was observed for these vehicles when compared with the neat solvents investigated. Hence, these results indicate PEG 400 as a useful tool to deliver the bioactive agents to the skin, instead of through the skin. According to the bioactive agent, skin retention may be more interesting than skin permeation. Skin retention of terbinafine was investigated by Ghose et al. through the design of polymeric nanosponge hydrogel. The antifungal agent was incorporated in Box–Behnken-design-optimized nanosponge formulations [8]. In vitro drug release from the nanosponge incorporated into the hydrogel was higher than the drug suspension or the marked formulation. Antifungal activity, nonirritancy, and no erythema or edema confirmed the promising application of the developed nanosponge hydrogel for efficient topical delivery of terbinafine hydrochloride.

Stimuli-responsive nanosystems have been designed to control the release of active molecules into the intended site of action. Van Gheluwe et al. applied a three-step synthesis of a redox-responsive blend of poly(ethylene glycol)–*block*–poly(lactide) (PEG–*block*–PLA) and poly(lactide) (PLA) to deliver retinol in the skin [9]. The selection of short-length polymers to incorporate the lipophilic active molecule allowed for achieving a high loading and rapid release of retinol. Stimuli responsiveness of the nanosystem was confirmed in vitro in the presence of L-glutathione. Good biocompatibility of black nanocarriers was observed in human keratinocytes, and low toxicity was detected in the presence of retinol. The redox-responsive blend of PEG–*block*–PLA and PLA were assembled by nanoprecipitation in smart nanocarriers able deliver other retinoid molecules for the treatment of skin diseases, like acne, photoaging, psoriasis vulgaris, melisma, and skin cancers. Cano-Cortes et al. investigated the drug covalent conjugation to the polymeric nanosystem based on PEGylated polystyrene pH-responsive polymer [10]. Doxorubicin was selected to evaluate this pH-responsive approach to cancer therapy. An efficient loading was achieved upon covalent conjugation of doxorubicin to cross-linked polystyrene nanoparticles, allowing selective drug release under acidic pH values. Breast and lung cancer cell lines were studied to determine the efficiency of cellular uptake, therapeutic activity, and genotoxicity effect. The pH-responsive polymeric nanosystems exhibited better antitumor activity in relation to free doxorubicin. The implemented chemical strategy could be further applied to other molecules and types of cancer.

## Data Availability

No new data were created or analyzed in this study. Data sharing is not applicable to this article.
